# Validation of a deep learning system for the detection of diabetic retinopathy in Indigenous Australians

**DOI:** 10.1136/bjo-2022-322237

**Published:** 2023-02-06

**Authors:** Mark A Chia, Fred Hersch, Rory Sayres, Pinal Bavishi, Richa Tiwari, Pearse A Keane, Angus W Turner

**Affiliations:** 1 Institute of Ophthalmology, University College London, London, UK; 2 Moorfields Eye Hospital NHS Foundation Trust, London, UK; 3 Lions Outback Vision, Lions Eye Institute, Nedlands, Western Australia, Australia; 4 Google Health, Palo Alto, California, USA; 5 Centre for Ophthalmology and Visual Science, The University of Western Australia, Nedlands, Western Australia, Australia

**Keywords:** Retina, Diagnostic tests/Investigation, Imaging

## Abstract

**Background/aims:**

Deep learning systems (DLSs) for diabetic retinopathy (DR) detection show promising results but can underperform in racial and ethnic minority groups, therefore external validation within these populations is critical for health equity. This study evaluates the performance of a DLS for DR detection among Indigenous Australians, an understudied ethnic group who suffer disproportionately from DR-related blindness.

**Methods:**

We performed a retrospective external validation study comparing the performance of a DLS against a retinal specialist for the detection of more-than-mild DR (mtmDR), vision-threatening DR (vtDR) and all-cause referable DR. The validation set consisted of 1682 consecutive, single-field, macula-centred retinal photographs from 864 patients with diabetes (mean age 54.9 years, 52.4% women) at an Indigenous primary care service in Perth, Australia. Three-person adjudication by a panel of specialists served as the reference standard.

**Results:**

For mtmDR detection, sensitivity of the DLS was superior to the retina specialist (98.0% (95% CI, 96.5 to 99.4) vs 87.1% (95% CI, 83.6 to 90.6), McNemar’s test p<0.001) with a small reduction in specificity (95.1% (95% CI, 93.6 to 96.4) vs 97.0% (95% CI, 95.9 to 98.0), p=0.006). For vtDR, the DLS’s sensitivity was again superior to the human grader (96.2% (95% CI, 93.4 to 98.6) vs 84.4% (95% CI, 79.7 to 89.2), p<0.001) with a slight drop in specificity (95.8% (95% CI, 94.6 to 96.9) vs 97.8% (95% CI, 96.9 to 98.6), p=0.002). For all-cause referable DR, there was a substantial increase in sensitivity (93.7% (95% CI, 91.8 to 95.5) vs 74.4% (95% CI, 71.1 to 77.5), p<0.001) and a smaller reduction in specificity (91.7% (95% CI, 90.0 to 93.3) vs 96.3% (95% CI, 95.2 to 97.4), p<0.001).

**Conclusion:**

The DLS showed improved sensitivity and similar specificity compared with a retina specialist for DR detection. This demonstrates its potential to support DR screening among Indigenous Australians, an underserved population with a high burden of diabetic eye disease.

**WHAT IS ALREADY KNOWN ON THIS TOPIC**

- Deep learning systems (DLSs) perform well at detecting diabetic retinopathy (DR) but can underperform in racial and ethnic minority groups, therefore external validation within these populations is critical for health equity. Indigenous Australians are a disadvantaged ethnic group who suffer disproportionately from diabetic eye disease.

**WHAT THIS STUDY ADDS**

- Compared with a retinal specialist, the DLS showed improved sensitivity and similar specificity for detecting DR in an Indigenous Australian population.

**HOW THIS STUDY MIGHT AFFECT RESEARCH, PRACTICE OR POLICY**

- Our study supports the potential of DLSs to improve retinopathy screening in the underserved Indigenous Australian population, although further work focusing on prospective validation and real-world implementation is required.

## Introduction

Diabetic retinopathy (DR) is the most common complication of diabetes and is among the leading causes of blindness in Australia.^1 2^ Indigenous Australians are disproportionately affected, suffering from more than five times the rate of diabetes-related vision impairment.^3 4^ Early detection and treatment through DR screening prevents vision loss in most cases, and there are clear international examples of where this has been achieved.^5^ Currently, almost half of Indigenous Australians are not receiving DR screening at the frequency recommended by national guidelines,^3^ in part due to insufficient availability of accessible and culturally appropriate services. With projected increases in the prevalence of diabetes, the provision of adequate DR screening services represents a major challenge for Australia.

Artificial intelligence (AI) algorithms for DR detection have shown promise in bridging the gap between demand and availability of screening resources, especially for underserved populations.^6^ Deep learning, a branch of AI particularly suited to image analysis, has enabled the development of systems that can rapidly and accurately detect DR on retinal photographs,^7–15^ without the need for referral to overburdened specialist services.

Despite generally performing well, an important limitation of deep learning systems (DLSs) is a tendency for reduced performance when applied to populations distinct from those in which they were developed.^16 17^ These discrepancies may arise for several reasons, such as variations in normal features or disease characteristics. Since the large training datasets required to develop a DLS tend to favour well-resourced populations, there are concerns that poor generalisability could lead to the exacerbation of healthcare inequities.^6^ Furthermore, there is evidence that existing structural biases may be translated into the performance of algorithms during training.^18^ Numerous examples exist within medical imaging where AI systems underperform among racial and ethnic minority groups.^19 20^ Recent work has demonstrated a possible mechanism for such a bias—DLSs learn to predict racial identity even when this is unrelated to the task at hand.^21^ Even more concerning, we are unable to prevent this from occurring since the basis for these predictions is unknown.^21^

The overall implication of these findings is that explicit assessment of model performance within racial and ethnic subgroups is critical.^20 21^ This is particularly important for disadvantaged communities where the benefits of improved efficiency are likely to have the greatest impact. This study aims to validate a DLS for the detection of DR among Indigenous Australians, an underserved population suffering disproportionately from diabetic blindness.

## Materials and methods

We performed a retrospective, external validation study comparing the performance of a DLS against a retina specialist for detecting DR from retinal photographs. This study follows the Standards for Reporting of Diagnostic Accuracy reporting guideline (online supplemental appendix A).^22^

10.1136/bjo-2022-322237.supp1 Supplementary data

### Algorithm overview

Our study applied the latest Conformité Européenne-marked version of a DLS designed for DR detection (indicates conformance with European Union product legislation). The algorithm’s development is described in detail by Krause *et al*.^8^ In brief, a deep neural network was trained with an ‘Inception-V.4’ architecture to predict a 5-point DR grade, referable diabetic macular oedema (DMO), and gradability for both DR and DMO. The input to the neural network was a colour retinal photograph with a resolution of 779×779 pixels. The neural network outputs a number between 0 and 1 (indicating its confidence) for each prediction. This value is determined through multiple computational stages, parameterised by millions of numbers.

The model was trained by presenting images from a training set consisting of 2.3 million retinal photographs with a known DR severity grade. For each photograph, the model predicted its confidence for the known severity grade, slowly adjusting its parameters to improve its accuracy over time. A tuning dataset evaluated the model throughout training to determine model hyperparameters. An ‘ensemble’ of five individual models was then created to combine predictions for the final output. To transform the model’s confidence-based outputs into discrete predictions, a threshold was used for each binary output (DMO, DR gradability and DMO gradability), and a cascade of thresholds was used to output a single DR severity level. Operating thresholds were optimised for high sensitivity suitable for a screening setting as previously described,^10^ and locked prior to the commencement of this study.

### Study population

This retrospective study was conducted at a single Aboriginal Community Controlled Health Service located within a metropolitan area of Perth, Western Australia. Participants were Aboriginal patients with diabetes attending a retinal screening service. Injection and laser treatment was available at monthly specialist clinics for patients identified by the screening service. The dataset consisted of retinal photographs acquired consecutively between July 2013 and October 2020. Images were non-mydriatic, single-field, 45°, macula-centred colour photographs captured using a Topcon Maestro retinal camera.

### Grading and adjudication

The DLS was compared against the performance of a single human grader selected from a pool of seven United States board-certified retina specialists (mean years of postfellowship experience: 5, range: 3–10). The specialist was provided with the same colour photograph as the DLS and asked to assess gradability for DR and DMO as indicated in online supplemental appendix B. For images deemed gradable for DR, the retina specialist applied the same 5-point International Clinical Diabetic Retinopathy (ICDR) severity scale,^23^ classifying images as no DR, mild non-proliferative DR (NPDR), moderate NPDR, severe NPDR or proliferative DR (PDR). For images deemed gradable for DMO, the retina specialist assessed the presence of referable DMO, defined as hard exudates within one disc diameter of the macula centre.^24^ Grades were applied using an online tool-based platform that has been previously described,^25^ based on disease characteristics from the ICDR severity scale. Graders were masked to the DLS and adjudication grades, and no additional clinical information was provided.

The reference standard consisted of a three-person adjudicated grade applied to all images by a panel of US board-certified retina specialists (mean 3.7 years postfellowship experience, range 1–6 years), using a method previously validated by Schaekermann *et al*.^25^ In brief, each adjudicating grader first performed an independent grade using the same online platform. Images demonstrating three-person agreement were considered resolved. For unresolved cases, images were reviewed by one panel member at a time in a round-robin fashion until agreement was reached. For each review round, the active grader reviewed previous grades and comments, regraded the given image and provided further comments as required.

### Outcome measures

For the primary outcomes, we combined individual assessments for gradability, DR severity and referable DMO to define the clinically relevant composite outcomes of more-than-mild DR (mtmDR), vision-threatening DR (vtDR) and all-cause referable DR. The definition of mtmDR was at least moderate NPDR or referable DMO. The definition of vtDR was at least severe NPDR or referable DMO. All-cause referable DR was defined as mtmDR or ungradable for mtmDR.

### Statistical analysis

We performed sample size calculations designed for use in diagnostic accuracy studies.^26^ We estimated a DLS sensitivity of 95% for detecting vtDR and set a minimum acceptable lower CI threshold of 90%. To achieve 95% confidence and 80% power, we required 183 eyes with vtDR. Assuming an ungradable rate of 15% and a vtDR prevalence of 15%,^11^ this resulted in a total required sample of 1440 diabetic eyes.

Statistical analysis was performed in IBM SPSS Statistics V.26. We generated 2×2 tables to characterise the sensitivity and specificity of the DLS and retina specialist (index tests) with respect to three-person adjudication (reference standard), at the eye level. The 95% CI for sensitivities and specificities were exact Clopper-Pearson intervals and p values were calculated using McNemar’s test. Quadratic-weighted Cohen’s kappa scores were calculated to measure agreement between the index tests and reference standard across the 5-point DR Scale.

## Results

### Participants

**Figure 1:**
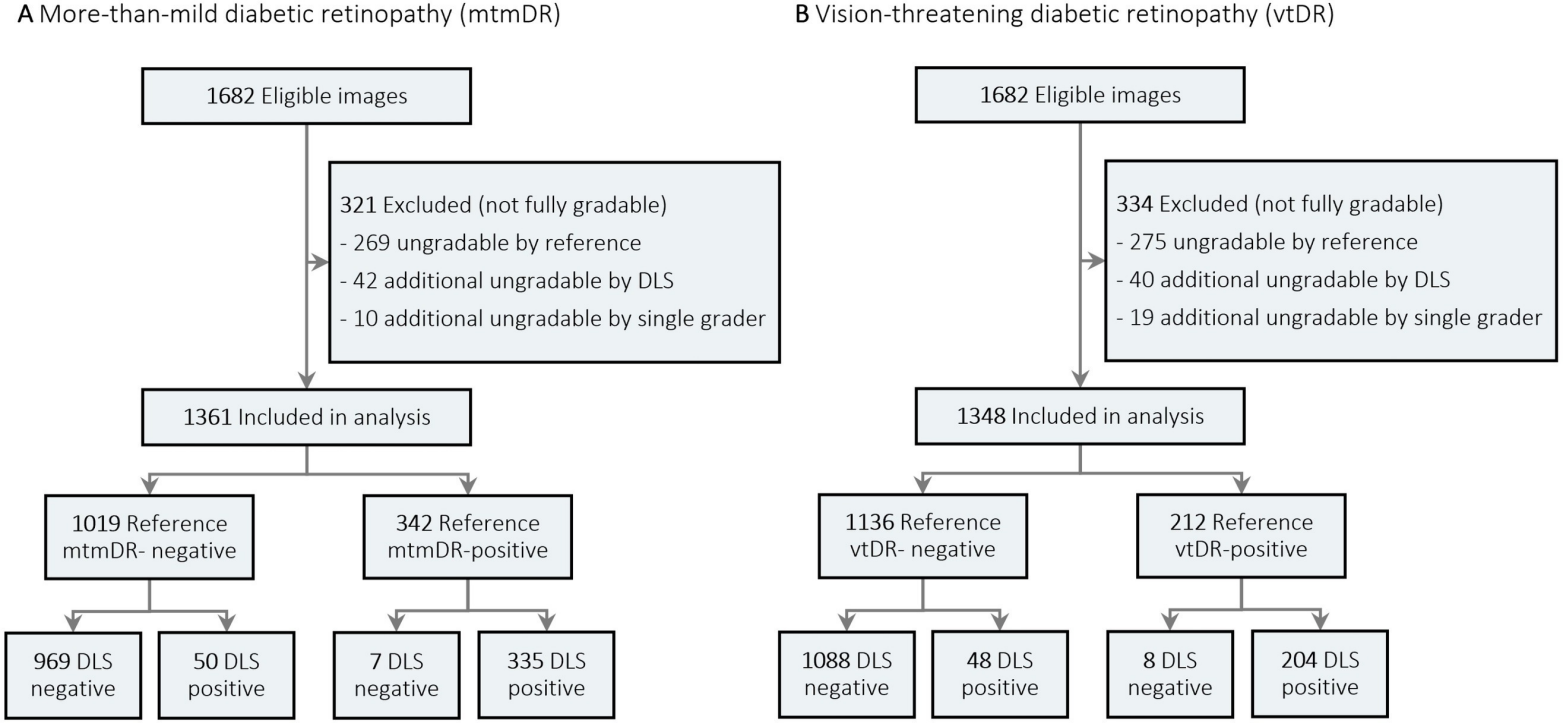
Flow diagram of image classification by reference standard and deep learning system (DLS). Differences in gradability arise since moderate non-proliferative diabetic retinopathy eyes that are ungradable for diabetic macular oedema are considered gradable for mtmDR but ungradable for vtDR.

Patient demographics and image characteristics of the external validation set are summarised in table 1. The validation set consisted of 1682 eyes of 864 patients. The mean age (SD) was 54.9 (15.0) years and women comprised 453 patients (52.4%). A flow diagram of image classification by the reference standard and DLS for mtmDR and vtDR is presented in figure 1. Of 1682 images, 1361 (80.9%) and 1348 (80.1%) images were included in the analysis for mtmDR and vtDR, respectively, with the remaining being ungradable by either the DLS, retinal specialist or reference standard.

**Table 1:** Baseline characteristics of Indigenous Australian dataset

Characteristic	n	%
Eyes (one image per eye)	1682	
Patient demographics		
Unique individuals	864	
Mean age, years (SD)	54.9 (15.0)	
Females	453	52.4
Diabetic retinopathy grade (eyes)		
None	1091	73.6
Mild	39	2.6
Moderate	260	17.5
Severe	11	0.7
Proliferative	82	5.5
Total gradable	1483	88.2
Diabetic macular oedema grade (eyes)		
Referable diabetic macular oedema	162	11.6
Total gradable	1391	82.7

### Performance

Sensitivities and specificities of the DLS and retina specialist for detecting mtmDR, vtDR and all-cause referable DR are summarised in table 2. The DLS had higher sensitivity compared with the retina specialist for detection of mtmDR (98.0% vs 87.1%, p<0.001), vtDR (96.2% vs 84.4%, p<0.001) and all-cause referable DR (93.7% vs 74.4%, p<0.001). Conversely, specificity of the DLS was lower than the retina specialist; however, this difference was small for mtmDR (95.1 vs 97.0%, p=0.006) and vtDR (95.8% vs 97.8%, p=0.002). The reduction in specificity was larger for all-cause referable DR (91.7% vs 96.3%, p<0.001). Quadratic-weighted kappa scores for the 5-point DR Scale were not substantially different for the DLS (88.0% (95% CI, 85.5 to 90.6)) and retina specialist (89.2% (95% CI, 86.7 to 91.6)). Confusion matrices for DR severity and referable DMO are presented in online supplemental appendix C.

**Table 2:** Comparison of deep learning system against a single retinal specialist for diabetic retinopathy detection, with reference to a three-person adjudication panel

	% (95% CI)*		P value†
	Deep learning system	Retinal specialist	
More-than-mild diabetic retinopathy‡			
Sensitivity	98.0 (96.5 to 99.4)	87.1 (83.6 to 90.6)	<0.001
Specificity	95.1 (93.6 to 96.4)	97.0 (95.9 to 98.0)	0.006
Vision-threatening diabetic retinopathy§			
Sensitivity	96.2 (93.4 to 98.6)	84.4 (79.7 to 89.2)	<0.001
Specificity	95.8 (94.6 to 96.9)	97.8 (96.9 to 98.6)	0.002
All-cause referable diabetic retinopathy¶			
Sensitivity	93.7 (91.8 to 95.5)	74.4 (71.1 to 77.5)	<0.001
Specificity	91.7 (90.0 to 93.3)	96.3 (95.2 to 97.4)	<0.001

*95% Exact Clopper-Pearson intervals.

†P value calculated between the deep learning system and retinal specialist using the McNemar test.

‡More-than-mild diabetic retinopathy (mtmDR) was defined as at least moderate non-proliferative diabetic retinopathy (NPDR) or diabetic macular oedema (DMO).

§Vision-threatening diabetic retinopathy was defined as at least severe NPDPR or DMO.

¶All-cause referable diabetic retinopathy was defined as mtmDR or ungradable for mtmDR.

### Gradability

The sensitivity for detecting ungradable cases of DR was higher for the DLS compared with the retina specialist (98.5% (95% CI, 96.5 to 100.0) vs 67.8% (95% CI, 61.3 to 74.4), p<0.001); however, specificity was lower (94.5% (95% CI, 93.5 to 95.8) vs 99.2% (95% CI, 99.8 to 99.6), p<0.001). For ungradable cases of DMO, the DLS showed higher sensitivity (66.7% (95% CI, 60.8 to 71.7)) vs 52.6% (95% CI, 45.7 to 57.7), p<0.001) and similar specificity (99.4% (95% CI, 98.9 to 99.8) vs 99.1% (95% CI, 98.6 to 99.6), p=0.48), although sensitivity was relatively poor for both. Confusion matrices for DR and DMO gradability are presented in online supplemental appendix C.

### Misclassification analyses

**Figure 2:**
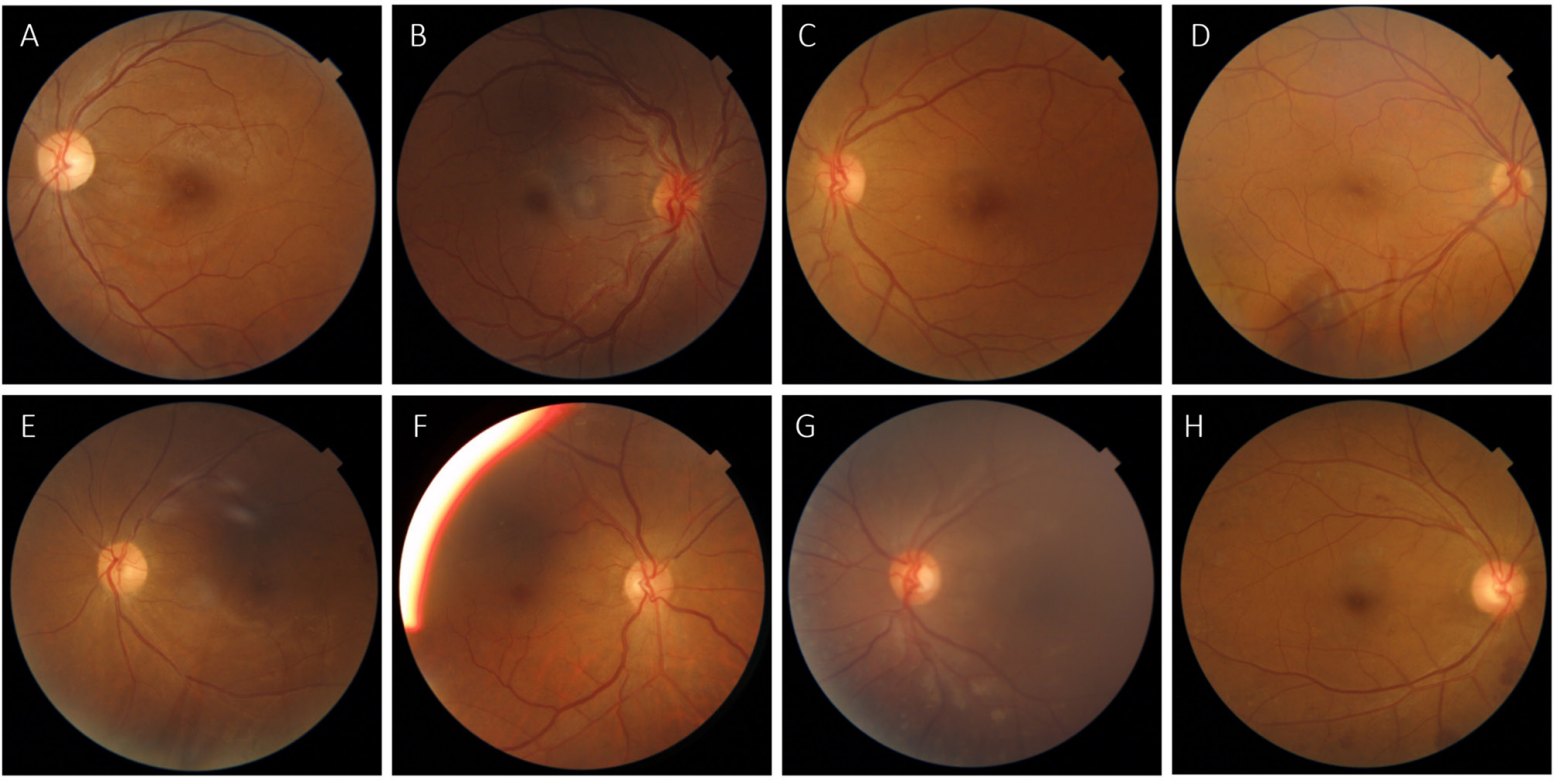
Retinal photographs of the 8/217 eyes diagnosed as vision-threatening diabetic retinopathy (vtDR) by the reference standard but missed by the deep learning system (DLS). According to the reference standard, A–F were graded as diabetic macular oedema (DMO), G was graded as proliferative diabetic retinopathy and H as severe non-proliferative diabetic retinopathy (NPDR). The DLS graded C as mild NPDR and the remainder as moderate NPDR, all without DMO. The single retinal specialist agreed with the DLS classification of no vtDR in all cases except D.

The DLS missed eight cases of vtDR (false negatives) according to the reference standard. All eight retinal photographs are shown in figure 2. These misclassifications comprised six cases of missed DMO, one case of missed PDR and one case of missed severe NPDR. The DLS identified mtmDR in all but one of these instances, indicating that cases would still have been referred but with less urgency (the remaining case was graded as mild DR). In seven out of eight cases, the single retina specialist agreed with the DLS classification of no vtDR rather than the reference standard, suggesting that these were likely difficult cases. The DLS also missed seven cases of mtmDR, which were all instead graded as mild DR. The single retina specialist agreed with the DLS in four of these instances, again suggesting borderline cases.

Of 53 eyes erroneously identified by the DLS as mtmDR (false positives), the DLS identified only moderate DR (the next lowest grade) in 37 (70%) cases. Of 53 eyes erroneously identified as vtDR (false positives), the reference standard result was mtmDR and therefore still referable in 37 (70%) cases. Inspecting the 5-point DR Scale confusion matrix (online supplemental appendix C), there were 10 cases in which the DLS predicted PDR but the reference standard concluded no DR. Of these, five cases had referable pathology identified in comments by the adjudication panel (three retinal vein occlusions, two disc oedemas), and a further four had clear referable pathology identified by an ophthalmologist (AT) during post-hoc misclassification analysis (adjudicators were not specifically advised to identify non-DR pathology). The remaining case exhibited a non-referrable vascular anomaly.

## Discussion

Our results demonstrate that the DLS was able to identify mtmDR and vtDR with performance similar to or exceeding a retina specialist in a cohort of Indigenous Australians. For the detection of mtmDR, vtDR and all-cause referable DR, sensitivity was considerably higher than the retina specialist. Although specificity was slightly reduced for mtmDR and vtDR detection, this trade-off would likely be considered acceptable within a typical screening setting, as missed cases have the potential to lead to poor visual outcomes.

For all-cause referable DR, the reduction in specificity was larger (91.7% vs 97.5%). This remains an important consideration when evaluating the viability of a screening programme due to the cost of false positive referrals. Of the all-cause referable DR errors made by the DLS, 53% were due to misclassifications between ‘no mtmDR’ and ‘ungradable for mtmDR,’ indicating that gradability disagreements were an important source of error. This is consistent with our findings of limited sensitivity for detecting ungradable DMO images by both the DLS (66.7%) and retina specialist (52.6%). Sensitivity for detecting ungradable images is often not consistently reported for DR detection systems.^12–15^ Reviewing the confusion matrices presented in Schaekermann *et al*,^25^ we noted there was poor agreement for DR gradability even between different three-person adjudication panels (mean sensitivity for detecting ungradable images was 44% across 12 comparisons). This finding implies that much of the reduction in performance for all-cause referable DR may arise due to poorly reproducible definitions of gradability, even among adjudication panels. Developing more consistent definitions of gradability may enable improved evaluation of DLS performance.

Kappa scores showed that agreement with the reference standard across the 5-point DR Scale was similar between the DLS and retina specialist. Importantly, while these scores penalise disagreements involving distant values from the reference, there is no additional penalisation for underestimating severity rather than overestimating severity. The DLS tended to overestimate severity compared with the retina specialist (online supplemental appendix C), which is generally a more acceptable error in a screening context. Misclassification analyses illustrated that DLS errors usually occurred in difficult or borderline cases. In most cases, these errors involved a misclassification to the adjacent category in the severity scale. Only eight cases of vtDR were missed and the single retina specialist agreed with the DLS in all but one of these instances.

This DLS has previously been applied to external validations sets in India^10^ and Thailand^9^ and results from our novel population group were comparable. For detecting moderate or worse DR in these studies, point estimates ranged between 88.9% and 96.8% for sensitivity and 92.2% and 95.6% for specificity. Reported performance for other DLSs for referable DR detection have ranged between 87.2% and 97.5% for sensitivity and 87.0% and 98.5% for specificity; however, definitions, study populations and methodology vary considerably.^16^

Our study has several strengths. First, the DLS was evaluated in a novel population suffering from a high burden of diabetic-eye disease. Second, classification thresholds were locked prior to the commencement of the study rather than being derived through post-hoc analysis of receiver operating curves. Third, we applied a consistent, rigorous reference standard to all images for external validation. Fourth, we report a range of composite outcomes that are clinically relevant to real-world screening programmes, including all-cause referable DR.

Our study has relevant limitations. Despite the use of a rigorous reference standard, we did not use optical coherence tomography imaging to define the presence of DMO, as has been recently described.^27^ The reference standard also did not include identification of non-DR referrable pathology. Although the DLS did identify important non-DR pathology in our misclassification analysis, it is possible that there was additional pathology that a retina specialist would have detected beyond the DLS. Our data came from a single centre, therefore our findings may not generalise to other Indigenous populations or to settings using alternative screening strategies such as multifield or dilated photography. Finally, as a retrospective study our validation set may not reflect the disease spectrum and challenges of a prospective cohort.

Future work should aim to address several challenges which remain for DLS-driven DR screening, with a focus on prospective validation and real-world implementation. Given the costs associated with false positive referrals using a fully automated model, the development of a hybrid model may provide a more practical option for implementation.^28^ This would involve the use of a DLS to rule out non-referable cases followed by secondary human assessment.

Careful consideration of processes for integrating DLSs into clinical-care pathways is critical, especially for Indigenous Australians. In addition to lower screening rates, Indigenous patients experience reduced follow-up after referral.^29^ Proposed explanations for this include: (1) higher proportions living in areas serviced by visiting specialists, (2) reduced accessibility through conventional communication pathways such as mail and telephone and (3) poor understanding of the need for attendance.^29^ A key benefit of a DLS is the ability to provide an immediate referral decision at the time of screening, facilitating in-person education and appointment planning. Although there is some supporting evidence derived from other settings that such a pathway would result in increased referral adherence,^30 31^ further work in this area is needed.

Prospective validation studies to date have identified relevant implementation challenges including poor internet availability and technical issues limiting consistent acquisition of gradable photographs.^32^ Large-scale deployment of a DLS for retinal screening is dependent on addressing these difficulties with validated solutions. In addition, it is known that a range of complex cultural factors influence the acceptability and uptake of healthcare interventions for Indigenous Australians, therefore collaboration with community leaders is essential.^29^ Fear and distrust towards Western medical practices is an important barrier to healthcare access in Indigenous communities, and it is possible that similar concerns may limit the uptake of AI-based solutions.

Our study shows that a DLS can detect DR in an Indigenous Australian cohort with improved sensitivity and similar specificity compared with a retina specialist. This demonstrates the potential of the system to support DR screening among Indigenous Australians, an underserved population with a high burden of diabetic eye disease. Inadequate DR screening represents an important source of healthcare inequity and is therefore an urgent priority for Australia.

## Data Availability

No data are available.
