# Supplementary material for: Validation of a deep learning system for the detection of diabetic retinopathy in Indigenous Australians
Source: Br J Ophthalmol. 2023 Feb 6;108(2):268–73. doi: 10.1136/bjo-2022-322237 (PMC10850716; doi:10.1136/bjo-2022-322237)
Supplement: Supplementary data [file bjo-2022-322237supp001.pdf]

Online-only Supplementary Material

Appendix A: STARD Checklist
Appendix B: Gradability Instructions and Examples
Appendix C: Confusion Matrices

Appendix A: STARD Checklist

Section & Topic	No	Item	Reported page #
**TITLE OR ABSTRACT**			
	**1**	Identification as a study of diagnostic accuracy using at least one measure of accuracy (such as sensitivity, specificity, predictive values, or AUC)	3
**ABSTRACT**			
	**2**	Structured summary of study design, methods, results, and conclusions (for specific guidance, see STARD for Abstracts)	3
**INTRODUCTION**			
	**3**	Scientific and clinical background, including the intended use and clinical role of the index test	6
	**4**	Study objectives and hypotheses	6
**METHODS**			
Study design	**5**	Whether data collection was planned before the index test and reference standard were performed (prospective study) or after (retrospective study)	8
Participants	**6**	Eligibility criteria	9
	**7**	On what basis potentially eligible participants were identified (such as symptoms, results from previous tests, inclusion in registry)	9
	**8**	Where and when potentially eligible participants were identified (setting, location and dates)	9
	**9**	Whether participants formed a consecutive, random or convenience series	9
Test methods	**10a**	Index test, in sufficient detail to allow replication	9
	**10b**	Reference standard, in sufficient detail to allow replication	9
	**11**	Rationale for choosing the reference standard (if alternatives exist)	9
	**12a**	Definition of and rationale for test positivity cut-offs or result categories of the index test, distinguishing pre-specified from exploratory	9
	**12b**	Definition of and rationale for test positivity cut-offs or result categories of the reference standard, distinguishing pre-specified from exploratory	N/A
	**13a**	Whether clinical information and reference standard results were available to the performers/readers of the index test	N/A
	**13b**	Whether clinical information and index test results were available to the assessors of the reference standard	9
Analysis	**14**	Methods for estimating or comparing measures of diagnostic accuracy	10
	**15**	How indeterminate index test or reference standard results were handled	10
	**16**	How missing data on the index test and reference standard were handled	10
	**17**	Any analyses of variability in diagnostic accuracy, distinguishing pre-specified from exploratory	N/A
	**18**	Intended sample size and how it was determined	10
**RESULTS**			
Participants	**19**	Flow of participants, using a diagram	Figure 1
	**20**	Baseline demographic and clinical characteristics of participants	Table 1
	**21a**	Distribution of severity of disease in those with the target condition	Table 1
	**21b**	Distribution of alternative diagnoses in those without the target condition	N/A
	**22**	Time interval and any clinical interventions between index test and reference standard	N/A
Test results	**23**	Cross tabulation of the index test results (or their distribution) by the results of the reference standard	Appendix
	**24**	Estimates of diagnostic accuracy and their precision (such as 95% confidence intervals)	Table 2
	**25**	Any adverse events from performing the index test or the reference standard	N/A
**DISCUSSION**			
	**26**	Study limitations, including sources of potential bias, statistical uncertainty, and generalisability	16
	**27**	Implications for practice, including the intended use and clinical role of the index test	17
**OTHER INFORMATION**			
	**28**	Registration number and name of registry	N/A
	**29**	Where the full study protocol can be accessed	N/A
	**30**	Sources of funding and other support; role of funders	18

2

Appendix B: Gradability Instructions and Examples

DR Gradability

How gradable is the image for DR? Note: This question doesn't show if "Other" was selected as Fundus field. Also, "Gradable" and "Gradable with Difficulty" were both considered as gradable images.

Gradable	• You can clearly see the features of DR in regions you'd expect to see in a given fundus field. This does not mean that you can confidently make a full diagnosis for DR with just this image.
Gradable with Difficulty	• Images show key regions for the defined field of view, but image quality is not good enough to allow for a confident grading<br>• Some key regions may be blurry or missing, but clearly visible regions show obvious pathology/features which point to at least moderate DR<br>• If visible regions don't show any pathology, then the image is "ungradable" as below
Ungradable	• Images don't show key regions with good enough quality for a confident grading. Also the other visible areas do not show any obvious pathology

DME Gradability

How gradable is the image for DME? Note: This question doesn't show if "Other" was selected as the Fundus field. Also, "Gradable" and "Gradable with Difficulty" were both considered as gradable images.

Gradable	• Entire macula (one disc diameter from fovea center) can be seen clearly. A confident diagnosis can be made.
Gradable with Difficulty	• Entire macula can be seen, but the image quality is not good enough to make a confident diagnosis.<br>• Part of the macula is missing, but there's strong evidence of DME (e.g hard exudates) in the visible area
Ungradable	• Macula is not visible or only partially visible (less than one disc diameter from fovea center) either because it is not in the field of view or because it is occluded by artifacts, dark shadow etc. What can be seen is not enough to rule out DME.<br>• DR symptoms are not clear in the image (ungradable for any symptom of DR) then mark image as ungradable for DME even though hard exudates are visible. (As hard exudates may not be due to Diabetic macular Edema.

Chia MA, *et al. Br J Ophthalmol* 2024; 108:268–273. doi: 10.1136/bjo-2022-322237

Examples

Images that were ungradable for diabetic retinopathy by the deep learning system and adjudicators.

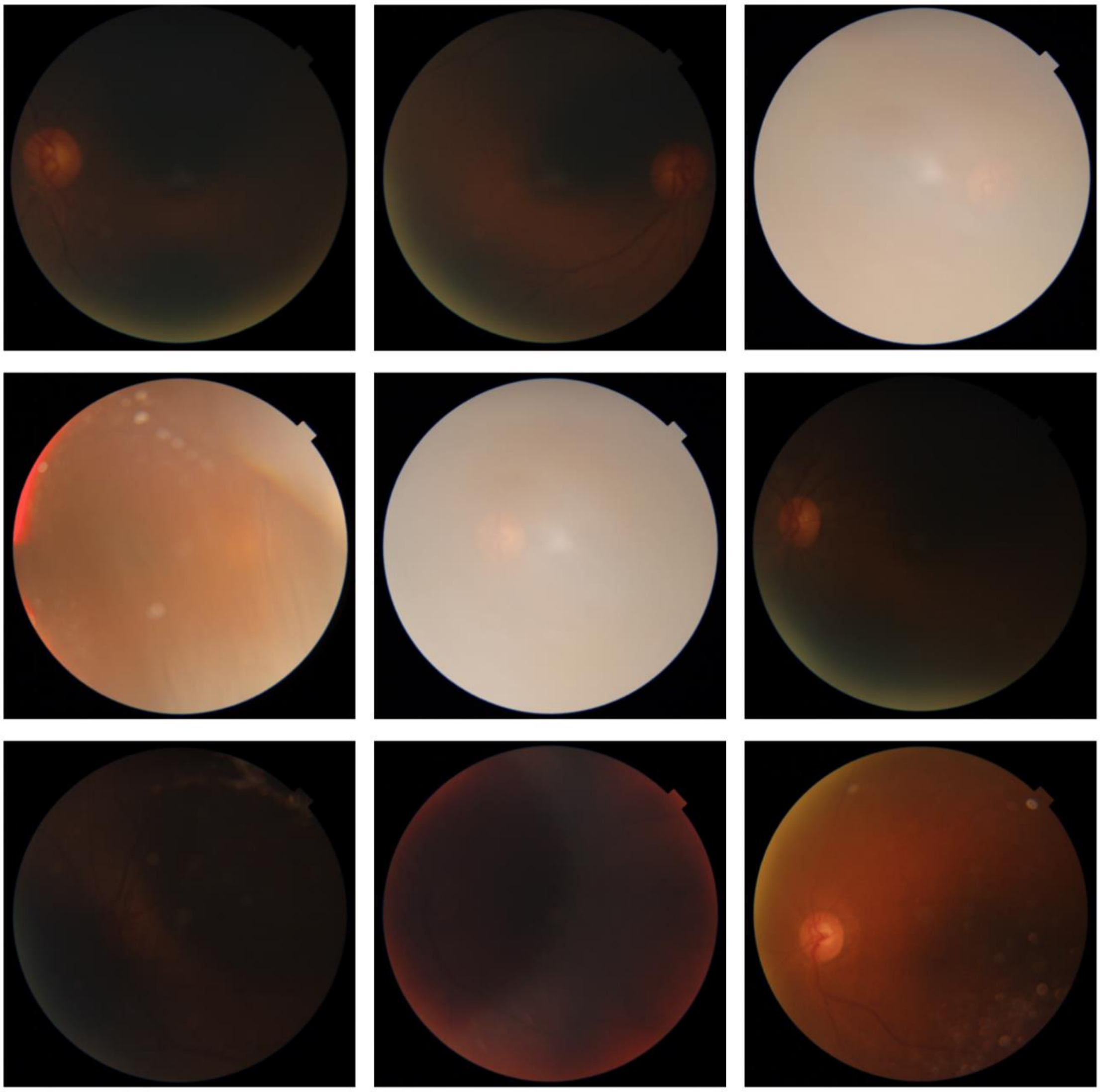

Appendix C: Confusion Matrices

DLS vs Reference - DR Confusion Matrix

		DLS						
		Ungradable	No DR	Mild NPDR	Mod NPDR	Severe NPDR	PDR	Total
Reference	Ungradable	196	1	1	0	0	1	199
	No DR	73	914	66	28	0	10	1091
	Mild NPDR	0	1	25	13	0	0	39
	Mod NPDR	6	1	7	150	70	26	260
	Severe NPDR	0	0	0	1	7	3	11
	PDR	2	0	0	1	0	79	82
Total		277	917	99	193	77	119	1682

Retina specialist vs Reference - DR Confusion Matrix

		Retina specialist						
		Ungradable	No DR	Mild NPDR	Mod NPDR	Severe NPDR	PDR	Total
Reference	Ungradable	135	51	1	11	0	1	199
	No DR	5	1047	18	20	1	0	1091
	Mild NPDR	1	13	22	3	0	0	39
	Mod NPDR	4	27	9	207	10	3	260
	Severe NPDR	0	0	0	8	3	0	11
	PDR	2	5	1	10	1	63	82
Total		147	1143	51	259	15	67	1682

DLS vs Reference - DME Confusion Matrix

		DLS			
		Ungradable	No DME	DME	Total
Reference	Ungradable	194	82	15	291
	No DME	7	1154	68	1229
	DME	1	7	154	162
Total		202	1243	237	1682

Retina specialist vs Reference - DME Confusion Matrix

		Retina specialist			
		Ungradable	No DME	DME	Total
Reference	Ungradable	153	130	8	291
	No DME	8	1192	29	1229
	DME	4	24	134	162
Total		165	1346	171	1682

DLS vs Reference - mtmDR Confusion Matrix

		DLS			
		Ungradable	No mtmDR	mtmDR	Total
Reference	Ungradable	231	32	6	269
	No mtmDR	35	972	53	1060
	mtmDR	7	7	339	353
Total		273	1011	398	1682

Retina specialist vs Reference - mtmDR Confusion Matrix

		Retina specialist			
		Ungradable	No mtmDR	mtmDR	Total
Reference	Ungradable	137	118	14	269
	No mtmDR	4	1034	22	1060
	mtmDR	6	42	305	353
Total		147	1194	341	1682

DLS vs Reference - vtDR Confusion Matrix

		DLS			
		Ungradable	No vtDR	vtDR	Total
Reference	Ungradable	233	35	7	275
	No vtDR	36	1097	53	1186
	vtDR	4	8	209	221
Total		273	1140	269	1682

Chia MA, *et al. Br J Ophthalmol* 2024; 108:268–273. doi: 10.1136/bjo-2022-322237

Retina specialist vs Reference - vtDR Confusion Matrix

		Retina specialist			
		Ungradable	No vtDR	vtDR	Total
Reference	Ungradable	141	125	9	275
	No vtDR	7	1160	19	1186
	vtDR	6	34	181	221
Total		154	1319	209	1682

DLS vs Reference – All-cause referable DR

Count

		DLS		
		Non-referable	Referable	Total
Reference	Non-referable	972	88	1060
	Referable	39	583	622
Total		1011	671	1682

Retina specialist vs Reference – All-cause referable DR

Count

		Retina specialist		
		Non-referable	Referable	Total
Reference	Non-referable	1034	26	1060
	Referable	160	462	622
Total		1194	488	1682

Note: Slight discrepancies exist when comparing matrices with Figure 1 and Table 2. Discrepancies arise due to the need to exclude images ungradable by any one of the reference, DLS, or retina specialist when performing pairwise statistical testing.

Chia MA, *et al. Br J Ophthalmol* 2024; 108:268–273. doi: 10.1136/bjo-2022-322237
